# Unidentified Flying Objects: An Infected Left Atrial Appendage Thrombus as a Nidus for Persistent Methicillin-Resistant Staphylococcus aureus Bacteremia

**DOI:** 10.7759/cureus.90387

**Published:** 2025-08-18

**Authors:** Mason Hemstreet, Jackson Carlyle, Warren S Hancock, Celia Campos Seegers, Bradley W Cantley

**Affiliations:** 1 Department of Research, Edward Via College of Osteopathic Medicine, Auburn, USA; 2 Department of Family Medicine, Edward Via College of Osteopathic Medicine, Auburn, USA; 3 Department of Family Medicine, Christ Health Center, Birmingham, USA; 4 Department of Physician Assistant Studies, Samford University, Homewood, USA

**Keywords:** infected thrombus, infective endocarditis, left atrial appendage (laa), mrsa bacteremia, septic embolization

## Abstract

Infections of intracardiac thrombi are exceptionally rare but pose significant clinical risks, especially when located in the left atrial appendage (LAA). We present a unique case of a 51-year-old male with multiple comorbidities who was found to have an infected LAA thrombus causing persistent methicillin-resistant *Staphylococcus aureus* (MRSA) bacteremia and systemic symptoms. Initial presentation included altered mental status, diabetic ketoacidosis, and classic signs of endocarditis such as splinter hemorrhages. Transesophageal echocardiogram imaging revealed a thrombus in the LAA, which was later confirmed to be infected and was acting as a nidus for sepsis. Surgical intervention and targeted antibiotic therapy led to clinical improvement. This case highlights the need for heightened clinical suspicion and a multidisciplinary approach when managing unexplained bacteremia, particularly in patients with known thrombotic risk factors. It also underscores the rare but significant potential for cardiac thrombi to become secondarily infected, warranting further study into diagnostic and therapeutic strategies.

## Introduction

The left atrial appendage (LAA) is a well-recognized site for thrombus formation, particularly in conditions such as atrial fibrillation. The LAA is the most common site of thrombus formation in atrial fibrillation because of its blind-ended, trabeculated anatomy, low flow velocities, and marked blood stasis, which together create ideal conditions for thrombogenesis [1-4]. LAA thrombus is present in 2-8% of patients with atrial fibrillation or atrial flutter undergoing transesophageal echocardiography, with a higher prevalence among those at increased stroke risk or presenting with acute stroke [5-8]. While this phenomenon is relatively common, a less familiar yet clinically significant complication is the concurrent infection of a thrombus within the LAA. Septic thrombi originating from the LAA are exceedingly rare, with only a scant number of case reports in the literature, making each presentation a valuable contribution to understanding this unusual entity. This rare occurrence can exacerbate the clinical course by introducing the risks of systemic disease, necessitating prompt recognition and management. While this condition is recognizable, the rarity of the added complication warrants further investigation and research.

## Case presentation

**Figure 1:**
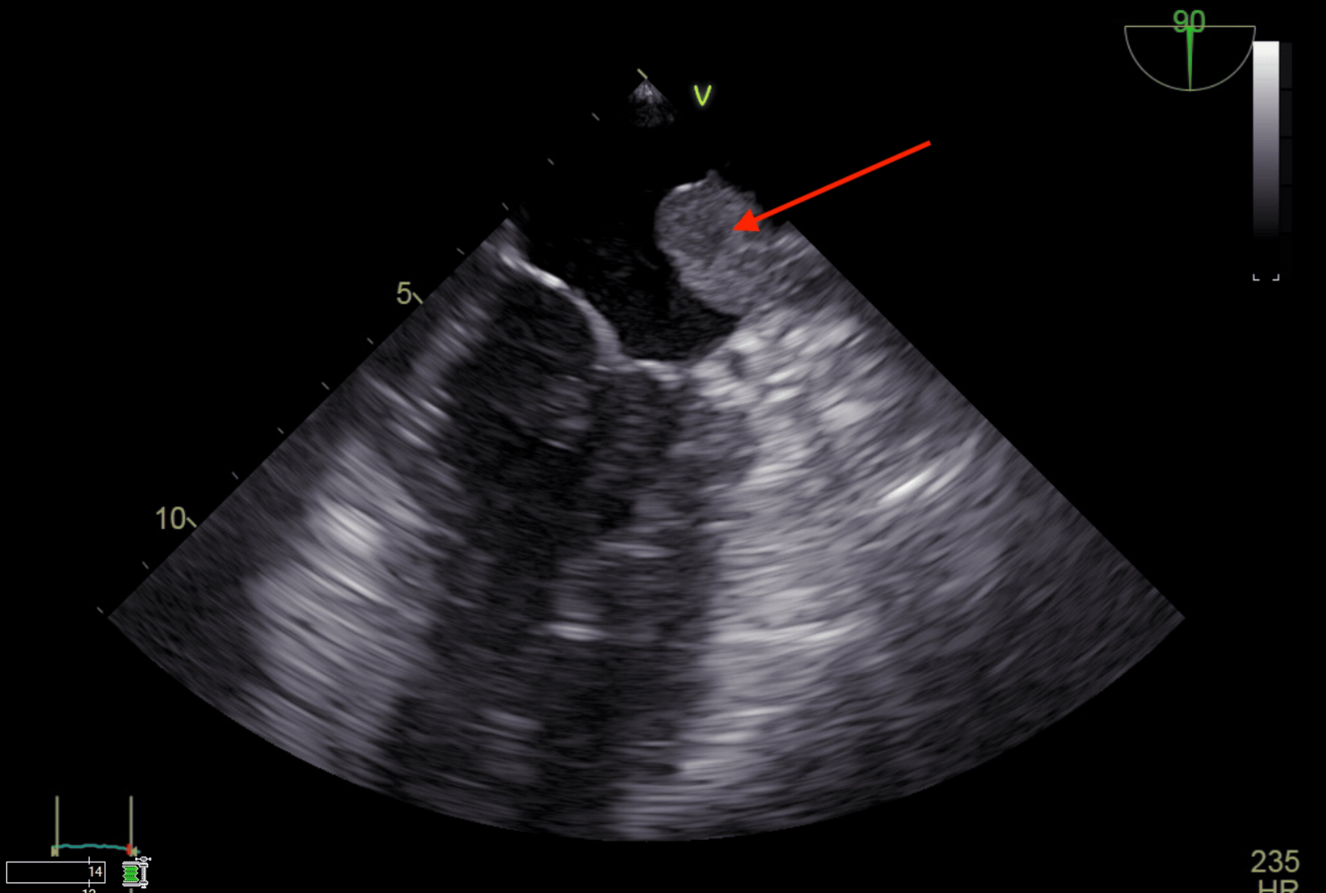
Transesophageal echocardiogram (TEE) showing an intracardiac thrombus inside the left atrium indicated by the red arrow

**Figure 2:**
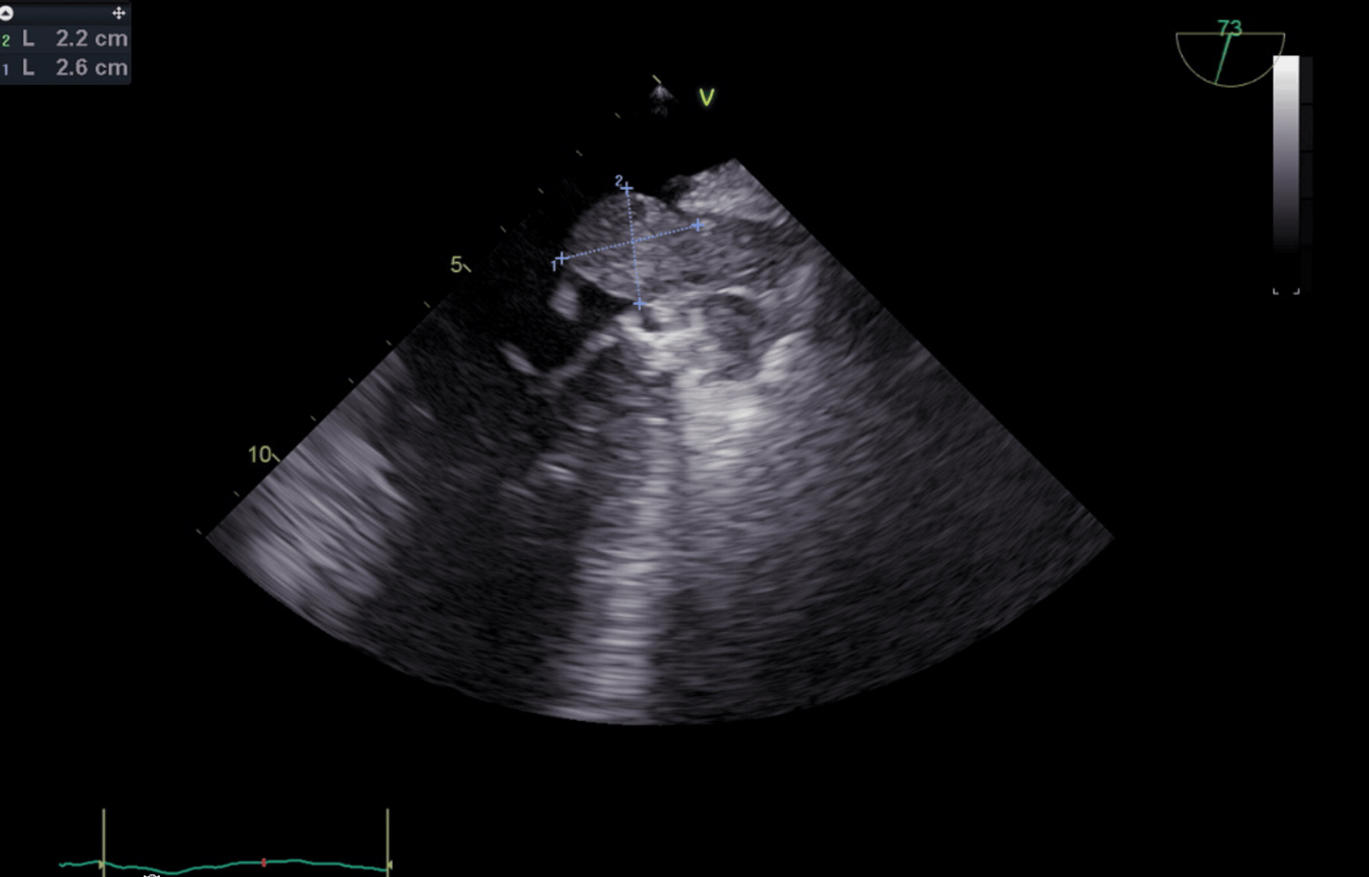
Transesophageal echocardiogram showing a 2.2 cm × 2.6 cm mass inside the left atrium indicated by the blue dashed lines

**Figure 3:**
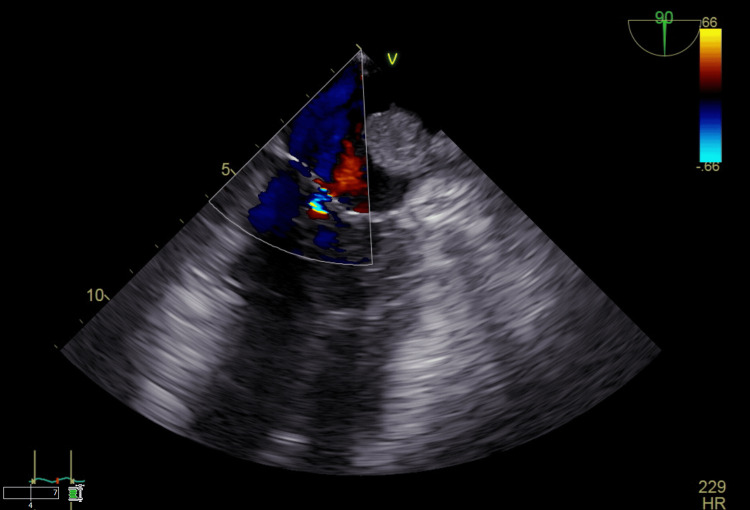
Transesophageal echocardiogram showing turbulent blood flow inside the left atrium

A 51-year-old male with a past medical history of type II diabetes mellitus, hyperlipidemia, past thrombotic events on anticoagulation, and coronary artery disease presented to the Emergency Department (ED) via emergency medical service (EMS) for unresponsiveness and hyperglycemia. According to the patient’s partner, the patient woke up in the middle of the night and was not acting like himself. One week before admission, the patient had been sick, exhibiting the symptoms of nausea, sweating, body aches, and excessive fatigue. Initially, the symptoms seemed to resolve, but recurred that night with altered mental status. The ED physician noted Kussmaul breathing, tachycardia, hypotension, point-of-care glucose of 600 mg/dL, and metabolic derangement. The patient was initially presumed to be in diabetic ketoacidosis and was started on large-volume resuscitation. A CT angiography (CTA) of the head/brain without contrast was ordered because of altered mental status and showed a 1.8 cm acute intraparenchymal hemorrhage in the left occipital region with no midline shift. A stat chest X-ray for the shortness of breath and Kussmaul breathing identified cardiomegaly with no pneumothorax or pleural effusion. A transthoracic echocardiogram was performed because of fever and suspicion of endocarditis from the classic vintage clinical pearl of splinter hemorrhages noted on the patient’s right ring finger and small pustules throughout the body. This showed an area of low attenuation at the mediastinum adjacent to the ascending aorta and the main pulmonary artery to the left. The ejection fraction was estimated to be 50-55% with mild mitral valve regurgitation, but vegetation could not be excluded. It was noted that there could be a complex fluid or a mass in the LAA. A thrombus was appreciated in the LAA extending into the left atrium, as seen in Figures 1-3.

Once the patient was stabilized, the providers at the hospital explored his history further. Of note, there was an incision and drainage with resection of the fourth and fifth metatarsal bones and arthrotomy with bone biopsy two years prior. There was no IV drug usage or alcohol consumption, but he had a smoking history of one pack per day. This was significant in the diagnosis of the patient because it was believed to the belief that this was the original cause of the patient’s bacteremia.

Two days after admission, a blood culture was positive for methicillin-resistant *Staphylococcus aureus* (MRSA), which led to a clinical discussion of where possible sepsis originated. Amputation of multiple right toes was performed five days after admission to the ED. No improvement was noted, which led to the belief that the LAA thrombus was causing septic issues. Eight days after admission, the LAA vegetation was removed, and the patient was treated for MRSA bacteremia secondary to endocarditis.

## Discussion

Three common differential diagnoses arise when a cardiac mass is visualized: vegetation, tumor, and thrombus [9]. However, what has been inadequately described in the literature, because of its rarity in clinical practice, is an intracardiac thrombus expanding into a vegetation and abscess via infection.

Infective endocarditis (IE) is defined as a bacterial infection of the inner heart lining or a heart valve [10]. A cardiac abscess is a suppurative infection that involves the endocardium and can expand into the myocardium [11]. Due to the friability of cardiac vegetations, septic emboli (SE) can occur and have been estimated in as high as 33% of IE cases, with increased mortality noted in IE complicated by SE [12]. Three physical exam findings related to septic embolization, described over a century ago, are Osler’s nodes, Janeway lesions, and splinter hemorrhages [13]. These are believed to occur due to the interplay between septic microemboli and the native immune system in the microvascular beds of the extremities [13].

An intracardiac thrombus is a blood clot that forms in the chambers of the heart [9]. One common location is the LAA, as this is an area where stagnation can occur in atrial fibrillation [14]. What is rather uncommon is that the infection of an atrial thrombus is rare. A case published in 2008 in the Journal of Cardiothoracic Surgery was reported to be only the second published case of an infected left atrial thrombus [15]. While infections of LAA closure device infections have been well documented, our review of the literature has only yielded one other case where an LAA thrombus served as a nidus for persistent bacteremia [16]. Furthermore, this case describes Osler’s nodes and splinter hemorrhages in a patient with persistent bacteremia and an infected LAA thrombus. This presents an unusual clinical phenomenon in which an intracardiac thrombus serves as both a nidus for clot embolization and infection.

Antibiotic therapy should be guided by the causative organism and its antibiotic susceptibility profile. For IE, the American Heart Association recommends specific antibiotic regimens based on the organism involved. For example, staphylococcal infection may require a combination of antibiotics such as vancomycin or daptomycin, often with rifampin and gentamicin if prosthetic material is involved [17]. In cases where the infection is stable and there is no paravalvular extension, a transition from intravenous to oral antibiotics may be considered, as supported by the POET trial, which demonstrated the noninferiority of oral step-down therapy in stable patients with left-sided IE [18,19]. However, the decision to switch to oral antibiotics should be made cautiously and typically involves consultation with infectious disease specialists.

## Conclusions

This case highlights the rare and complex occurrence of an infected LAA thrombus, a condition that can complicate the clinical course by introducing both thrombotic and infectious risks. While infections of cardiac masses are well-documented, the occurrence of thrombus infection within the LAA is seldom described in clinical practice. Prompt recognition and intervention are crucial, as the infection can lead to systemic complications such as bacteremia and septic embolization.

The clinical management of infected LAA thrombus requires a multidisciplinary approach, involving cardiology, infectious disease, and surgical teams. Early detection is key, as delays in treatment can lead to severe complications, including septic emboli, organ damage, and increased mortality. In this case, the timely diagnosis and removal of the infected thrombus, combined with targeted antibiotic therapy, stabilized the patient’s condition. Persistent bacteremia despite adequate source control should prompt consideration of an intracardiac thrombus infection, particularly in patients with risk factors such as atrial fibrillation and diabetes. Surgical removal of the infected thrombus along with pathogen-directed antibiotics can be curative, underscoring the importance of maintaining a high index of suspicion in similar scenarios.
